# Supplementary material for: COVID-19 Vaccination Intentions amongst Healthcare Workers: A Scoping Review
Source: Int J Environ Res Public Health. 2022 Aug 17;19(16):10192. doi: 10.3390/ijerph191610192 (PMC9407815; doi:10.3390/ijerph191610192)
Supplement: Supplementary file 1 [file ijerph-19-10192-s001.zip › ijerph-1864571-supplementary.pdf]

Table S1: Search terms – Variants of keywords used in the database search.

KEYWORDS			
**COVID-19**	**Healthcare personnel**	**Vaccination attitudes**	**Intellectual disability**
"Wuhan coronavirus*" OR "COVID19*" OR "COVID-19*" OR "COVID-2019*" OR "coronavirus disease 2019" OR "SARS-CoV-2" OR "2019-nCoV" OR "2019 novel coronavirus" OR "severe acute respiratory syndrome coronavirus 2" OR "2019 novel coronavirus infection" OR "coronavirus disease 2019" OR "coronavirus disease-19" OR "SARS-CoV-2019" OR "SARS-CoV-19"	"healthcare prof*" OR "healthcare work*" OR "health personnel" OR "healthcare staff" OR "caregiver"	"vaccine" OR "immunisations" OR "vaccination" OR "vaccination schedule" AND "belief" OR "perceptions" OR "views" OR "attitudes" OR "opinions" OR "knowledge"	"intellectual disability" OR "learning disability"
